# Identification and Characterization of a Phenyl(trifluoro-methyl)-pyrimidine Positive Allosteric Modulator of the Secretin Receptor

**DOI:** 10.3390/membranes16070249

**Published:** 2026-07-21

**Authors:** Kaleeckal G. Harikumar, Daniela G. Dengler, Leire Borrega Roman, Robert Ardecky, Eduard A. Sergienko, Laurence J. Miller

**Affiliations:** 1 Department of Molecular Pharmacology and Experimental Therapeutics, Mayo Clinic, Scottsdale, AZ 85259, USA; 2 Conrad Prebys Center for Chemical Genomics, Sanford Burnham Prebys Medical Discovery Institute, La Jolla, CA 92037, USA

**Keywords:** secretin receptor, class B GPCR, positive allosteric modulator

## Abstract

G protein-coupled receptors (GPCRs) are among the most common drug targets, with numerous agonists and antagonists approved for clinical use. More recently, it has been appreciated that drugs can also modulate the action of natural agonists of these receptors, thus providing unique clinical advantages. Here, we describe the identification and characterization of a small molecule positive allosteric modulator (PAM) of secretin action at the class B G protein-coupled secretin receptor. This phenyl(trifluoromethyl)-pyrimidine can occupy the secretin receptor without stimulating its internalization, yet priming it to enhance both the potency and efficacy of the action of natural secretin. This is also shown to exhibit its effects on cells expressing low numbers of these receptors, without enhancing the effects of other structurally related hormones acting at other class B GPCRs. The mechanism responsible for this PAM effect is the slowing of the off-rate of receptor-bound secretin. This compound can serve as a lead to the development of other drugs that enhance the action of natural endogenous secretin and can be utilized to explore the potential therapeutic utility of such compounds.

## 1. Introduction

The class B G protein-coupled secretin receptor is important for the stimulation of hepatobiliary and pancreatic duct cells, cardiac myocytes, vascular smooth muscle cells, pancreatic islet cells, adipocytes, and selected neurons [1]. Secretin hormonal action has the classical effect of stimulating alkaline secretion into the upper small bowel to neutralize acidic chyme leaving the stomach, and it has been described to have potential uses in managing heart failure [2,3], hypertension [4], obesity [5,6], diabetes [5], GI gastrointestinal dysmotility states [7], and some forms of cancer [8,9]. The natural peptide agonist for this receptor is utilized clinically as a diagnostic when used in short intravenous infusions during radiographic studies of the biliary tract [10] and to test for gastrin-secreting islet cell tumors [11], but no clinically useful therapeutic agents to activate this receptor have yet been approved for clinical use.

We previously described an in vitro high-throughput effort to identify potential small molecule agonists and positive allosteric modulators of the secretin receptor [12]. This screening effort utilized cell lines expressing a wild-type human secretin receptor to test compounds in a small molecule library for their ability to stimulate cAMP, as well as their ability to modulate secretin-stimulated cAMP responses. We also tested the ability of compounds to modulate the activity of less potent non-naturally-occurring analogs of secretin at the secretin receptor, in an effort to enhance the sensitivity of the assay. We reported five promising chemical scaffolds for small molecule ligands of the secretin receptor having agonist and/or allosteric modulator activity at the secretin receptor. We subsequently reported the first candidate probes for these activities coming from this campaign [13,14]. Early probes with PAM activity that we identified in that report were non-selective, also acting at the GLP-1 receptor [14]. Reliance on our robust testing funnel [12] has now also resulted in our identification of a new promising lead candidate not previously reported, which possesses positive allosteric modulatory activity to selectively enhance secretin action at the secretin receptor. This molecule has a unique chemical structure that is the focus of the current report.

## 2. Materials and Methods

### 2.1. Materials

#### 2.1.1. Receptor Ligands

A phenyl(trifluoromethyl)-pyrimidine, SBI-344 (N-(3-chloro-4-methoxy phenyl)-5-(4-methylphenyl)-7-(trifluoromethyl)-4H,5H,6H,7H-pyrazolo[1,5-a]pyrimidine-2-carboxamide; Molport-007-920-671), was identified as a lead positive allosteric modulator of secretin action at the secretin receptor (SecR) in the application of a previously described screening effort [12]. Human secretin(1-27) and fluorescent secretin analogue, secretin(1-27)-Gly^28^(Cys^29^-Alexa^488^), were prepared and purified in our laboratory [15]. Human secretin(3-27) and secretin(1-23) were custom synthesized (GeneScript Biotech, Piscataway, NJ, USA). Human vasoactive intestinal polypeptide (VIP), calcitonin (CT), gastric inhibitory polypeptide (GIP), glucagon-like peptide-1 (GLP-1), and glucagon (GCG) were purchased from American Peptide Company (Sunnyvale, CA, USA).

#### 2.1.2. Cell Lines

Chinese hamster ovary (CHO) cells and NG108-15 cells [16] were purchased from the American Type Culture Collection (ATCC) (Manassas, VA, USA). Human secretin receptor-bearing CHO cells (hSecR and low hSecR (lower level of SecR expression)) and rat secretin receptor-bearing CHO cells (rSecR) were previously established and characterized [17]. CHO-VPAC1R, CHO-GLP-1R, and CHO-CTR cells were previously established and characterized [18,19,20]. CHO-GCGR and CHO-GIPR cell lines were prepared for this project in analogous fashion. Flp-in-T-Rex cells bearing SNAP-tagged SecR WT cells were previously prepared and characterized [21].

#### 2.1.3. Reagents

The HTRF cAMP dynamic assay kit was from Revvity (Boston, MA, USA), and SNAP-surface-Alexa^488^ dye was from NEB Labs (Ipswich, MA, USA). Fluo-8-AM was from AAT Bioquest Inc. (Sunnyvale, CA, USA). Fetal clone II and tissue culture supplements were from Life Technologies (Carlsbad, CA, USA). Probenecid and 3-isobutyl-1-methylxanthine were from Millipore-Sigma (St Louis, MO, USA). All other reagents were of analytical grade.

### 2.2. Methods

#### 2.2.1. cAMP Accumulation Assays

cAMP was quantified in 96-well plates with the homogeneous time-resolved fluorescence (HTRF) cAMP Gs dynamic kit, applied as we previously described [22]. In these assays, compound SBI-344 was studied alone to examine its intrinsic agonist activity, as well as in the presence of secretin to examine its potential allosteric action. SecR-expressing cells were stimulated with concentrations (0 to 10 µM) of full-length secretin(1-27) peptide, and truncated secretin analogs, secretin(3-27), and secretin(1-23), in the presence or absence of compound SBI-344 (10 µM) to examine possible positive allosteric modulatory (PAM) activities. Data were analyzed using the operational model of allosterism described in Kenakin and Christopoulos [23] and Wootten et al. [24] to determine the cooperativity factors. Cells were seeded at a density of 20,000 cells/well 24 h prior to the assay. Assays were started by washing the cells with PBS, pH 7.4, and stimulated with agonists by mixing with Kreb’s-Ringers-HEPES (KRH) medium (25 mM HEPES, pH 7.4, 104 mM NaCl, 5 mM KCl, 2 mM CaCl_2_, 1 mM KH_2_PO_4_, and 1.2 mM MgSO_4_, with 0.01% soybean trypsin inhibitor and 0.2% bovine serum albumin) supplemented with 0.1% bacitracin and 1 mM 3-isobutyl-1-methylxanthine for 30 min at 37 °C. Incubations were terminated by aspiration of the medium, and cells were lysed with 6% perchloric acid for 15 min by shaking. The pH of the cell suspension was adjusted to 6.0 with 30% KHCO_3_. Cell lysates were used for quantifying the cAMP response following the manufacturer’s instructions. cAMP responses were measured by a time-resolved FRET protocol (excitation (Ex) 337 nm, with emission (Em) measured at both 620 and 665 nm; calculating the ratio of data from 650 nm/620 nm) using the PheraSTAR FSX with software version 5.41 (BMG LabTech Inc., Cary, NC, USA). Data were analyzed and plotted using Prism 10.2. This assay was also utilized to examine the specificity of agonist activity at other structurally related class B GPCRs, as well NG108-15 cells.

#### 2.2.2. Calcium Response Assays

Intracellular calcium levels were determined in secretin receptor-expressing cells as described previously [22]. Cells were grown to approximately 70–80% confluence on 96-well black clear bottom culture plates and incubated in the dark with 0.75 µM Fluo 8AM in KRH medium containing 2.5 mM probenecid and 0.2% bovine serum albumin for 1 h at 37 °C. Cells were washed twice with KRH medium and stimulated with increasing concentrations of secretin in the presence or absence of compound SBI-344 (10 µM) at 37 °C in Flexstation 3 (Molecular Devices, Sunnyvale, CA, USA). All assays were performed in duplicate and repeated a minimum of three times in independent experiments. Peak responses were determined from the fluorescence emission intensities at 525 nm after exciting the samples at 485 nm over a period of 120 s. The times when peak responses occurred were not significantly different for different conditions. Responses were quantified and reported as percentages of the peak responses to 100 µM ATP. The concentration-response data were plotted using a non-linear log (agonist) vs. response curve with three-parameter curve fitting using Prism 10.2.

#### 2.2.3. Fluorescence Polarization Assay

Fluorescence polarization (FP) was utilized to evaluate the binding kinetics of secretin peptide to receptor-bearing membranes isolated from CHO-SecR cells [25]. The assay was performed using secretin(1-27)-Gly^28^(Cys^29^-Alexa^488^) in a Pherastar FSX instrument (BMG Labtech, Cary, NC, USA), following the fluorescence anisotropy protocol (Ex 480 nm, Em 520), with measurements read for 0.5 s/cycle. Non-specific signals were measured in the presence of a saturating concentration of secretin peptide throughout the protocol. Binding kinetics were initiated by adding secretin(1-27)-Gly^28^(Cys^29^-Alexa^488^) in the absence or presence of the compound (SBI-344, 10 µM) to secretin receptor-bearing membranes to a final volume of 200 µL of binding buffer (KRH buffer, pH 7.4 with 0.2% bovine serum albumin) in a 96-well black Opti-plate (Perkin Elmer) for a minimum of 75 cycles. After reaching a plateau, dissociation of the fluorescent ligand from the receptor was initiated by adding a saturating concentration of unlabeled secretin (1 µM) in the absence or presence of compound SBI-344 (10 µM) (at 50 cycles) and collecting the FP signal for another 25 cycles. The final kinetic data were calculated using non-linear regression curve fitting with association and then dissociation parameters with maximal iterations of fitting using Prism 10.2.

#### 2.2.4. SecR Internalization Assay

We studied the ability of compound SBI-344 to stimulate SecR internalization, compared with internalization stimulated by the natural agonist peptide, full length secretin. Flp-in-TREX cells stably expressing SNAP-tagged WT SecR were used for these studies [13]. Cells grown on polylysine-coated coverslips had receptor expression induced with tetracycline (1 µg/mL) for 24 h before labeling. Cells were washed with phosphate buffer saline (PBS), pH 7.4, containing 0.2% bovine serum albumin, followed by incubation with 3 µM SNAP-surface-Alexa488 dye (NEB Labs, Ipswich, MA, USA) for 30 min at 37 °C. Cells were washed twice with PBS and incubated further with either secretin or the compound (SBI-344) in PBS, pH 7.4, supplemented with CaCl_2_ and MgCl_2_ for 2 h at 4 °C. After incubation, the cells were washed with PBS and incubated with PBS at 37 °C for different time periods, as indicated. Cells were fixed with 2% paraformaldehyde solution, then mounted on coverslips using Vectashield. Cell surface fluorescence was acquired using an inverted microscope (40×) controlled by QED InVivo software, version 3.03 (Media Cybernetics, Bethesda, MD, USA). Cell surface fluorescence in three independent experiments was quantified using Image J (NIH). Representative fluorescence images were assembled using Photoshop CC 2018.

#### 2.2.5. Statistics

Comparisons between experimental conditions and controls were evaluated using the Mann–Whitney test. Values of *p* < 0.05 were considered to be statistically significant.

## 3. Results

**Figure 1:**
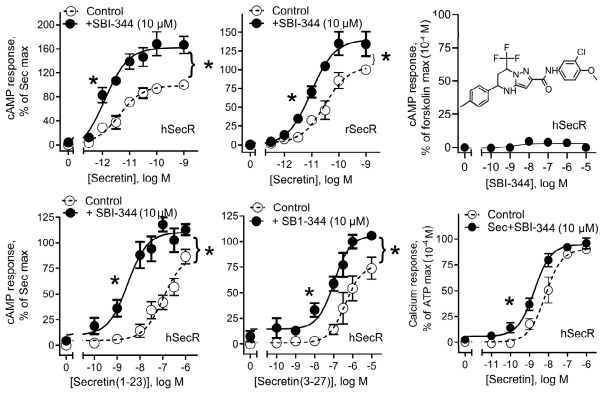
Agonist-dependent biological responses in SecR-expressing cells. Shown are the dose-dependent cAMP responses in human SecR-expressing cells by the full-length natural peptide ligand, secretin(1-27) (**Top Left**), and truncated peptide ligands, secretin(1-23) (**Bottom Left**) and secretin(3-27) (**Bottom Middle**), rat SecR (**Top Middle**) in the presence and absence of SBI-344 (10 µM). Shown also are secretin dose-dependent intracellular calcium responses (**Bottom Right**) in the presence and absence of SBI-344 (10 µM). Shown is the intrinsic agonist action of compound in human SecR expressing cells (**Top Right**). Values are expressed as means ± S.E.M. from 4 independent experiments performed in duplicate. The structure of SBI-344 is shown in the inset (**Top Right**). Statistical analyses were performed using the Mann–Whitney test (* *p* < 0.05 significantly different from control).

The ability of SBI-344 to act as a positive allosteric modulator of secretin action at SecR is shown in Figure 1, with EC_50_ and E_max_ data shown in Table 1. This compound exhibited no intrinsic ability to stimulate cAMP in human SecR-bearing cells in concentrations as high as 10 µM (Figure 1, top right panel), while it was able to significantly enhance the ability of natural secretin to stimulate cAMP responses in human and rat SecR-bearing cells (Figure 1, top left and center panels). Both the EC_50_ and the maximal responses to secretin were significantly affected by this compound. Similar enhancement of effects of N- and C-terminally truncated non-naturally occurring reduced potency analogs of secretin at the human SecR were observed (Figure 1, bottom left and center panels). This provided confirmation of the modulatory activity. There was also significant enhancement of the EC_50_ of secretin-stimulated calcium responses mediated through Gq, rather than Gs, the dominant coupling mechanism (Figure 1, bottom right panel).

**Figure 2:**
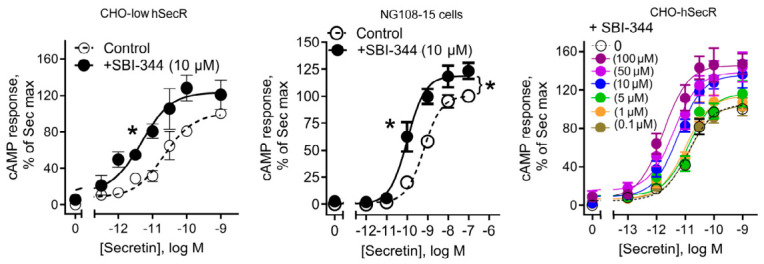
Secretin-dependent cAMP responses in SecR-expressing cells. Shown are the secretin dose-dependent cAMP responses in low-level SecR-expressing cells (**Left** panel) and NG108-15 neuroblastoma cells that naturally express SecR (**Middle** panel) in the absence and presence of SBI-344 (10 µM). Shown also is the dose-dependent positive allosteric modulation of SBI-344 (**Right** panel). Values are expressed as means ± S.E.M. from 4–8 independent experiments performed in duplicate. Statistical analyses were performed using the Mann–Whitney test (* *p* < 0.05 significantly different from control).

The positive allosteric modulatory effect of SBI-344 was similarly demonstrated with the CHO cell line expressing lower numbers of SecR (Figure 2, left panel) (21 ± 3 × 10^3^ sites/cell for this line versus 67 ± 2 × 10^3^ sites/cell for the standard CHO-SecR cell line used in the previous studies), as well as the NG108-15. hybrid neuroblastoma/glioma cells naturally expressing SecR in a density (13 ± 3 × 10^3^ sites/cell) similar to the low expressing CHO cell line [16] (Figure 2 center panel).

The allosteric constants for SBI-344 were calculated based on the data presented in the right panel of Figure 2 and Table 2 in which increasing concentrations of this compound modulated the biological activity of the natural full agonist, secretin, using the operational model of allosterism described in Kenakin and Christopoulos [23] and Wootten et al. [24]. In this calculation, K_b_ and τ_b_ are the dissociation constant (affinity) and intrinsic efficacy of the allosteric compound, with α and β the cooperativity factors for this compound to affect the affinity and efficacy of secretin. Logαβ > 0 indicates positive allosterism, while Logαβ = 0 and Logαβ < 0 indicate neutral cooperativity and negative allosterism, respectively. Logαβ of 1.4 ± 0.2 (αβ = 25) in Table 2 indicates substantial positive cooperativity, reflecting the significant positive allosteric modulatory influence of SBI-344 on the secretin biological activity.

**Figure 3:**
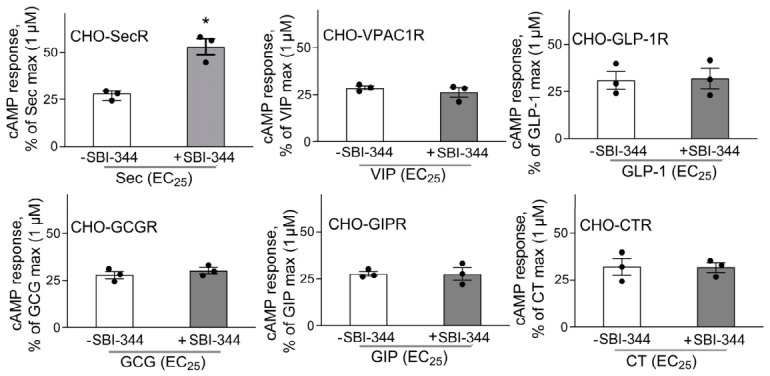
Specificity of compound action on other family B receptors. Shown are bar graphs reflecting natural ligand-induced cAMP responses on EC_25_ concentrations of natural peptide ligands (the concentration of the natural agonist ligand of that receptor that stimulates 25% of its maximal signal in the absence of modulator) in the absence and presence of SBI-344 (10 µM). CHO-SecR (**Top Left**), CHO-VPAC1R (**Top Middle**), CHO-GLP-1R (**Top Right**), CHO-GCGR (**Bottom Left**), CHO-GIPR (**Bottom Middle**), CHO-CTR (**Bottom Right**), Values are expressed as means ± S.E.M. from 3 independent experiments performed in duplicate. Statistical analyses were performed using the Mann–Whitney test (* *p* < 0.05 significantly different from control).

The PAM action of SBI-344 to enhance the response to secretin at SecR exhibited specificity, with no analogous action at a selected series of other structurally related class B GPCRs (Figure 3). The activity studied for each receptor was that of the action of its natural agonist, using an approximate EC_25_ concentration to stimulate 25% of that agonist’s maximal effect in the absence of a modulator.

**Figure 4:**
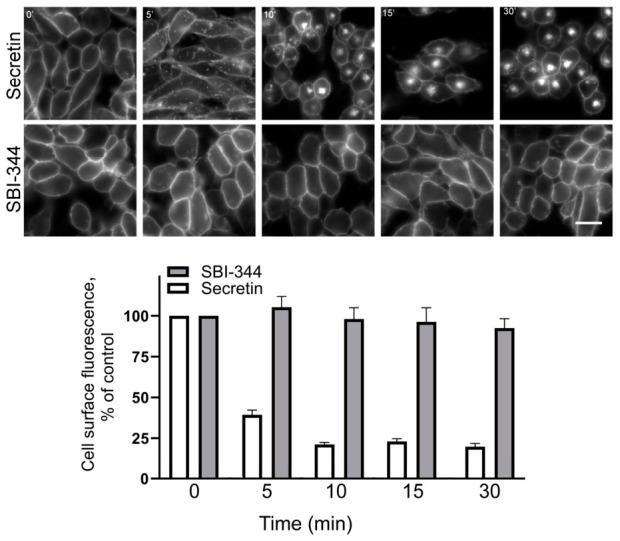
Effect of the compound on secretin receptor internalization. Shown are representative fluorescence microscopic images of time-dependent SecR trafficking after stimulation with either secretin (1 nM) or SBI-344 (10 µM). Cell surface fluorescence was quantified using Image J in three independent experiments, and graphed below as percentages of the values at the beginning of the experiments. The secretin peptide stimulated receptor internalization in a time-dependent manner, while the compound did not stimulate receptor internalization. Scale bar (25 µm) shown here.

Figure 4 shows the ability of the natural agonist, secretin (1 nM), to stimulate the internalization of SecR, while SBI-344 (10 µM) did not exhibit this activity. This is an important characteristic for a modulator such as SBI-344, allowing the receptor to remain on the cell surface where the natural agonist hormone would have access to it when it is released physiologically. If the PAM stimulated receptor internalization, it would no longer be able to respond to the natural agonist.

**Figure 5:**
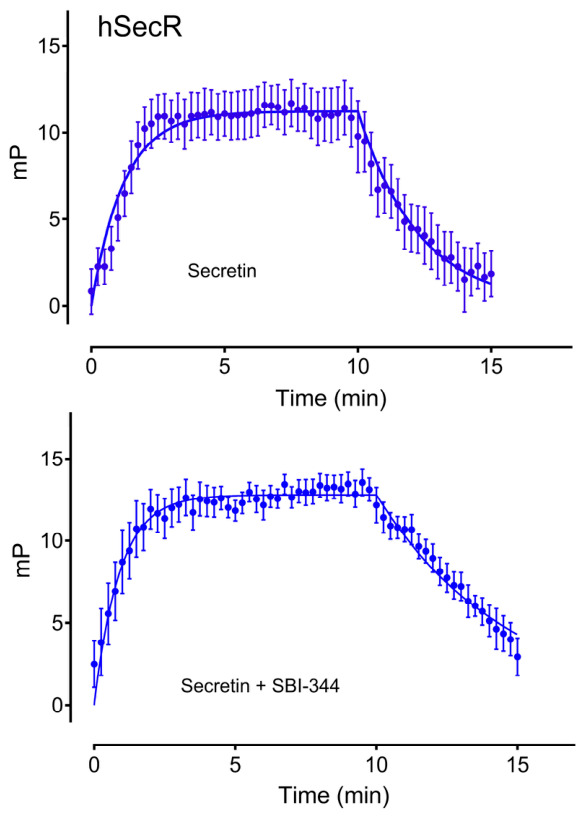
Kinetic binding profile of secretin(1-27)-Gly^28^,(Cys29-Alexa^488^) in the absence and presence of SBI-344. Shown are the kinetic binding profiles of secretin association and dissociation to SecR-expressing cell membranes in the absence or presence of compound (10 µM). Values are expressed as means ± S.E.M. from 4 independent experiments performed in triplicate.

Figure 5 shows the impact of SBI-344 on the association (the initial upslope of the curves) and dissociation (the later downslope of the curves) kinetics of secretin binding to SecR. The kinetic data are quantified in Table 3. Only the rate of secretin dissociation was significantly affected by SBI-344, slowing this event, such that the duration of receptor occupation with secretin was prolonged. This explains the PAM activity of this compound.

## 4. Discussion

While secretin was the first hormone to be discovered, described in 1902 [26], with its receptor recognized as a prototypic class B GPCR [27], this hormone-receptor system has not been a prominent target for drug development until recently. The natural secretin peptide has been utilized during short infusions as a diagnostic reagent, to stimulate pancreatico-biliary secretion, useful in evaluating pancreatic exocrine insufficiency and sphincter of Oddi dysfunction [10], as well as the identification of gastrin-secreting islet cell tumors [11]. Secretin-like agents with potential therapeutic utility have not yet been approved for clinical use.

Class B GPCRs have been challenging targets for small molecule drug development, since they possess an open intrahelical bundle without distinct pockets such as are commonly targeted by small molecule ligands in class A GPCRs. There are now many peptide ligands being utilized to target this class of GPCRs [28]. There are also finally orally active small molecule agents targeting related GPCRs that are being developed and that are entering the clinic [29]. We recognized this possibility and began a high-throughput screening program for small molecule agonists and PAMs of the secretin receptor several years ago [12]. This resulted in our recent report of small molecule thiadiazole agonists of this receptor [13]. We also identified several scaffolds in that series that possessed PAM activity but that were not specific, also enhancing GLP-1R function [14]. We now report a promising lead for a specific small molecule secretin receptor PAM with encouraging pharmacologic properties.

SBI-344 is a phenyl(trifluoromethyl)-pyrimidine that enhances the action of endogenous secretin to stimulate cAMP and calcium accumulation in secretin receptor-bearing cells, active both in high expressing model systems and in low receptor-expressing models and endogenous receptor-bearing cells. This compound also has similar effects at the rat secretin receptor, making it useful in pre-clinical testing. It has this action despite exhibiting no intrinsic agonist activity. It is also capable of occupying the secretin receptor without stimulating receptor internalization, thus priming the cell for its positive allosteric modulatory action on endogenous secretin. The positive allosteric modulatory action of SBI-344 on secretin biological activity is shown to be mediated by slowing the rate of dissociation of bound secretin.

Allosteric modulators have been shown to have potentially unique pharmacologic properties [30], such as being able to only exhibit biological action at the time and location of action of the endogenous agonist hormone. This can limit their impact to effects on a physiologically relevant event. They can shift the efficacy and/or potency of the endogenous agonist/hormone in a setting in which that response might be insufficient or excessive.

The secretin receptor is expressed on numerous cell types, including hepatobiliary and pancreatic duct cells, cardiac myocytes, vascular smooth muscle cells, pancreatic islet cells, adipocytes, and selected neurons, as well as pathologic cells, such as some tumors [31]. Potential uses of secretin agonists have been described, such as in the management of heart failure, hypertension, obesity, diabetes, GI dysmotility states, and receptor-expressing tumors [30]. It is not clear whether there would be an advantage or disadvantage of limiting action in such settings to only the finite periods of time when the endogenous agonist/hormone might be secreted. Now, with the availability of agents with this PAM activity, this question can be addressed.

## Figures and Tables

**Table 1:** Effect of SBI-344 on agonist-induced biological responses in SecR-expressing cell lines.

Cell Lines	Ligands	pEC_50_	n, *p* Values	E_max_	*p* Values
*cAMP responses*					
CHO-hSecR	Sec(1-27) + SBI-344	11.3 ± 0.2 11.9 ± 0.1 *	7, 0.03	97.8 ± 3.2 148.5 ± 16.3 *	0.03
CHO-hSecR	Sec(1-23) + SBI-344	7.0 ± 0.3 8.5 ± 0.2 *	4, 0.03	64.1 ± 11.0 102.8 ± 4.3 *	0.03
CHO-hSecR	Sec(3-27) + SBI-344	6.3 ± 0.1 7.4 ± 0.4 *	5, 0.03	79.5 ± 13.4 114 ± 10.5	0.11
CHO-rSecR	Sec(1-27) + SBI-344	10.3 ± 0.1 11.2 ± 0.1 *	4, 0.03	93.3 ± 3.9 126.0 ± 14.1	0.06
NG108-15	Sec(1-27) + SBI-344	9.2 ± 0.1 10.0 ± 0.1 *	4, 0.03	97.7 ± 3 120.8 ± 8.6 *	0.03
*Calcium responses*					
CHO-hSecR	Sec(1-27) + SBI-344	8.0 ± 0.1 8.9 ± 0.1 *	4, 0.03	87.5 ± 1.3 94.0 ± 4.1	0.11

Values are expressed as means ± S.E.M. from 4–7 experiments performed in duplicate. Statistical analyses were performed using the Mann–Whitney test (* *p* < 0.05 significantly different from control).

**Table 2:** Allosteric constants for the impact of SBI-344 on secretin-stimulated cAMP responses in SecR cells.

Parameters	Sec + SBI-344
pK_b_	3.7 ± 0.3
Logτ_b_	1.1 ± 0.2
Logαβ (αβ)	1.4 ± 0.2 (25)
n	8

Values are expressed as means ± S.E.M. from 8 experiments performed in duplicate.

**Table 3:** Kinetic parameters of secretin ligand association and dissociation in the absence/presence of SBI-344.

	Secretin	Secretin + SBI-344	*p* Values
K_on_ × 10^8^ M^−1^ min^−1^	0.8 ± 0.3	1.4 ± 0.1	0.19
K_off_ × M^−1^	0.5 ± 0.1	0.2 ± 0.1 *	0.03
pKi	8.0 ± 0.3	8.8 ± 0.1	0.10
n	3	3	

Values are expressed as means ± S.E.M. from 3 experiments performed in duplicate. Statistical analyses were performed using the Mann–Whitney test (* *p* < 0.05 significantly different from control).

## Data Availability

The raw data supporting the conclusions of this article will be made available by the authors on request.

## Abbreviations

The following abbreviations are used in this manuscript:

GPCR	G protein-coupled receptor
PAM	Positive allosteric modulator
SecR	Secretin receptor
VIP	Vasoactive intestinal polypeptide
GIP	Gastric inhibitory polypeptide
GLP-1	Glucagon-like peptide-1
GCG	Glucagon
